# ssvQC: an integrated CUT&RUN quality control workflow for histone modifications and transcription factors

**DOI:** 10.1186/s13104-021-05781-8

**Published:** 2021-09-20

**Authors:** Joseph Boyd, Princess Rodriguez, Hilde Schjerven, Seth Frietze

**Affiliations:** 1 Department of Biomedical and Health Sciences, College of Nursing and Health Sciences, University of Vermont, Burlington, VT USA; 2 Cellular Molecular Biomedical Sciences Program, University of Vermont, Burlington, VT 05405 USA; 3 Department of Laboratory Medicine, University of California, San Francisco, CA 94143 USA; 4 The University of Vermont Cancer Center, Burlington, VT 05405 USA

**Keywords:** CUT&RUN, ChIP-seq, Data quality control, Data visualization

## Abstract

**Objective:**

Among the different methods to profile the genome-wide patterns of transcription factor binding and histone modifications in cells and tissues, CUT&RUN has emerged as a more efficient approach that allows for a higher signal-to-noise ratio using fewer number of cells compared to ChIP-seq. The results from CUT&RUN and other related sequence enrichment assays requires comprehensive quality control (QC) and comparative analysis of data quality across replicates. While several computational tools currently exist for read mapping and analysis, a systematic reporting of data quality is lacking. Our aims were to (1) compare methods for using frozen versus fresh cells for CUT&RUN and (2) to develop an easy-to-use pipeline for assessing data quality.

**Results:**

We compared a workflow for CUT&RUN with fresh and frozen samples, and present an R package called ssvQC for quality control and comparison of data quality derived from CUT&RUN and other enrichment-based sequence data. Using ssvQC, we evaluate results from different CUT&RUN protocols for transcription factors and histone modifications from fresh and frozen tissue samples. Overall, this process facilitates evaluation of data quality across datasets and permits inspection of peak calling analysis, replicate analysis of different data types. The package ssvQC is readily available at https://github.com/FrietzeLabUVM/ssvQC.

**Supplementary Information:**

The online version contains supplementary material available at 10.1186/s13104-021-05781-8.

## Introduction

The genome-wide profiling of chromatin-associated proteins and posttranslational modifications (PTMs) to histones has revolutionized research in epigenetic gene regulation [1–3]. One of the mainstay techniques used to map the enrichment patterns of PTMs and proteins is ChIP-seq, in which proteins are physically crosslinked to target DNA, immunoprecipitated with specific antibodies, and their crosslinks are reversed for downstream deep sequencing and analysis of enriched DNA [4, 5]. ChIP-seq assays typically require a large amount of starting material in the range of millions of cells per reaction, and depending on the antibody, can result in a high amount of background. A recently developed method termed Cleavage Under Targets and Release Using Nuclease (CUT&RUN) is becoming widely employed in laboratories for mapping the genomic interactions of proteins [6, 7]. CUT&RUN is an in situ genome-wide profiling method that employs an antibody-targeted micrococcal nuclease (MNase) fusion protein to selectively digest and release DNA fragments at protein binding sites. Overall, this method results in considerably lower background compared to ChIP-seq [7], and can be employed using much less starting material [8].

While multiple tools exist for CUT&RUN data processing and peak calling [9, 10], there is a need for software to conduct CUT&RUN data quality control (QC), which should be performed prior to detailed data analysis and interpretation. To increase the reproducibility and reliability of the data, a detailed evaluation of the data is essential when performing the assays in different sources of biological material and when testing new antibodies. In the event of a low-quality assay (high background or low signal), a comparative QC process is vital to understanding possible sources of error to adjust experimental conditions and improve data quality.

Here we introduce ssvQC as a framework for CUT&RUN data quality assessment. We use this tool to evaluate cell storage protocols for performing CUT&RUN from a limited number of cells and demonstrate that frozen cells from spleen can be used to obtain consistent genome-wide enrichment profiles for transcription factor and histone modifications generated by CUT&RUN. Overall, ssvQC integrates a set of useful QC metrics to reliably summarize data quality and can be implemented as a user-friendly R package.

## Main text

### Material and methods

#### Ethics statement

Animal experiments were approved by University of California San Francisco animal research (# AN177290) and University of Vermont animal research ethics committees (IUACUC# PROTO201900021) and were carried out in accordance with approved guidelines.

#### Mice

C57BL/6 mice were obtained from The Jackson Laboratory (Bar Harbor, USA). Mice (6–8 weeks old) of both genders were used for experiments. The animals were bred and maintained either at the animal facilities at University of California San Francisco, or at the University of Vermont. Animals were sacrificed by CO_2_ asphyxiation followed by cervical dislocation.

#### Magnetic B cell enrichment

Spleens were harvested and single-cell suspensions were prepared. Cells were washed once with PBS and red blood cells were lysed with ACK lysis buffer (150 mM NH_4_Cl, 10 mM KHCO_3_, 0.1 mM Na_2_EDTA). B cells were isolated using MACS (Miltenyi Biotech, cat # 130–090-862). Purities of enriched B cells of > 90% were determined by flow cytometry. Freezing was performed by resuspending cell pellets in 10% DMSO/90% FBS and then slowly frozen at -1 °C/minute.

#### CUT&RUN assays

CUT&RUN was performed on fresh (2–8 million cells) or frozen cells (0.5 million cells). CUT&RUN libraries were built using published protocols (EpiCypher, Skene and Henikoff, 2017) with some modifications. Nuclei was isolated from fresh cells using hypotonic lysis buffer (20 mM HEPES–KOH pH 7.9, 10 mM KCl, 1 mM MgCl_2,_ 0.1% Triton X-100, 20% Glycerol) and then washed (20 mM HEPES pH 7.5, 150 mM NaCl, 0.5 mM Spermidine, 2 mM EDTA, 0.1% BSA). Frozen cells were thawed for 2 min in a 37 °C water bath and washed twice (20 mM HEPES, pH 7.5, 150 mM NaCl, 0.5 mM Spermidine, 1 × Roche cOmplete, EDTA-free protease inhibitor). Nuclei were bound with concanavalin A coated magnetic beads (Polysciences) and incubated overnight with primary antibody at 4 °C. Unbound antibody was washed and proteinA/G-MNAse (EpiCypher) was added and activated with CaCl_2_ added to a final concentration of 2 mM for 3 h at 4 °C and quenched with 2X Stop buffer (340 mM NaCl, 20 mM EDTA, 4 mM EGTA, 50 µg/mL RNaseA, 50 µg/mL glycogen). Samples were incubated at 37 °C for 20 min and placed on a magnet to release protein-DNA fragments. Supernatant containing fragments was transferred to a new tube and the final volume was raised to 300 µl. To extract DNA, 15 µl of DNA extraction buffer (3 µl of 10% SDS (final concentration 0.1%), 5 µl of proteinase K (at 10 mg/ml), 2 µl RNaseA (at 1 mg/ml), and 5 µl of 5 M NaCl (final concentration 300 mM)) was added, vortexed, and incubated at 50 °C for 1 h. DNA was purified by phenol/chloroform/isoamyl alcohol extraction and ethanol precipitation with glycogen. Libraries were constructed using the NEBnext Ultra II DNA Library Prep Kit (Illumina) as directed with modifications. To retain fragments > 150 bp after adapter ligation, a 1.1 × AMPure XP bead cleanup (Beckman Coulter) was performed. Libraries were PCR amplified for 14 cycles (1 cycle: 45 s at 98 °C, 14 cycles: 15 s at 98 °C followed by 10 s 60 °C, 1 cycle: 1 min at 72C). The DNA was precipitated with 1.1 × volume of AMPure XP beads and fragment size distribution was assessed via Bioanalyzer (Agilent). Samples were pooled and sequenced on HiSeq platform using paired end sequencing. The antibodies used in this study were: rabbit anti-H3K4me3 (EpiCypher, cat 13–0041; lot no 20083002–42; 0.5 μg); rabbit anti-Ikaros (Santa Cruz, sc-13039; 0.5 μg) and rabbit anti-IgG (Epicypher, cat 13–0042; lot no 20036001–52; 0.5 μg).

#### Data processing, peak calling and analysis

For data preprocessing, we used the CUT&RUN tools pipeline as described [10].

#### Implementation of ssvQC

ssvQC is implemented in R and can run on both MacOS, Windows, and Linux (bigWig operations will not work on Windows). The package can be found at https://github.com/FrietzeLabUVM/ssvQC. The tool is flexible to use and can be run with the minimal inputs of several bam files and corresponding peak files or can be fully controlled via a plain text configuration file. Data processing operations are implemented in parallel where possible for speed and results are cached to prevent redundant work. All plot outputs are ggplot objects to allow easy customization and intermediate tidy formatted data objects are easily accessible to allow complete control by users. A complete QC report is outputted as a CSV file.

### Results

**Fig. 1:**
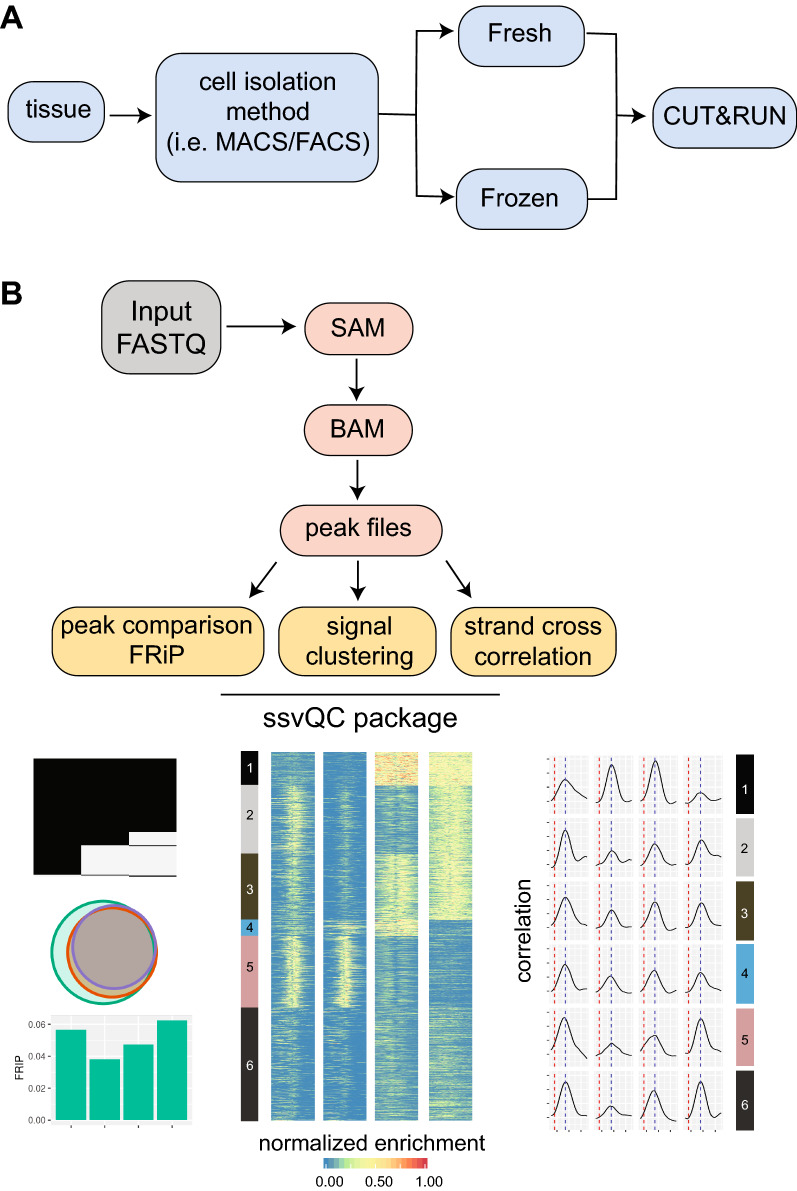
Experimental and data analysis workflows to evaluate CUT&RUN from fresh and frozen samples. **A** Experimental process used to compare fresh and frozen cell isolates for CUT&RUN analysis for histone modification and transcription factors. **B** Data analysis workflow for ssvQC package. Arrows show the order of the steps. Data preprocessing steps are indicated in salmon color and ssvQC output files are grouped in yellow color. Shown are representative ssvQC output files, including summaries of overlapping peak sets via binary heatmap or Euler plots, clustered heatmap of CUT&RUN signal at peak regions, and strand cross correlation (SCC) analysis showing estimated fragment length (blue line) and read length (red line)

To study the gene regulatory mechanisms of B cells, we aimed to generate genome-wide datasets for regulatory histone modifications and transcription factors (TFs) from a mature B cell population isolated from fresh mouse spleen tissue. Because cell isolation from tissue can be time consuming and the downstream processing of fresh material is not always possible, we wanted to evaluate if isolated cells could be frozen using a slow freezing protocol (see methods) for CUT&RUN assays and how the results would compare to that of fresh material (Fig. 1A). We therefore performed a pilot CUT&RUN experiment using antibodies specific for the histone modification H3K4me3, the TF Ikaros and control IgG, using fresh or frozen cells.

To assess the overall data quality, we implemented a uniform data processing pipeline and developed a quality control package called ssvQC to systematically collect and represent CUT&RUN data quality metrics. The overall data processing workflow is illustrated in Fig. 1B, showing the preprocessing steps leading up to the input data files for ssvQC. The output of preprocessing steps includes mapped BAM files, a signal BigWig file, and the significantly enriched regions in narrowPeak, broadPeak and SEACR bed file format. We also implemented a routine to upload signal files to UCSC genome browser for generating shared sessions, facilitating sharing of data. ssvQC can similarly be applied to other sequence enrichment assays, including ChIP-seq and ATAC-seq.

**Fig. 2:**
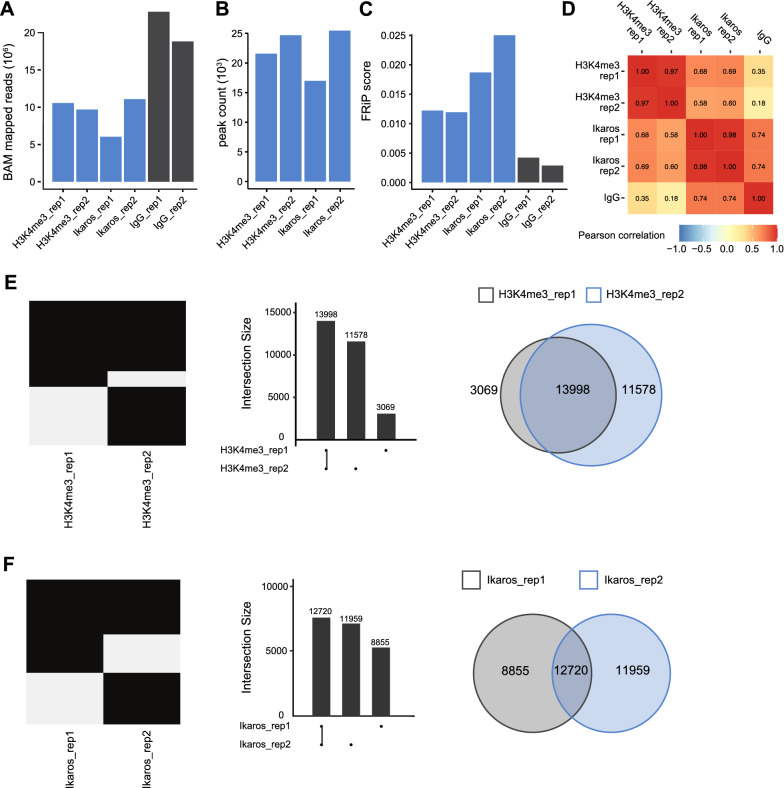
Evaluation of CUT&RUN peaks for transcription factors and histone modifications with replicates using the ssvQC package. The ssvQC output plots for H3K4me3 and Ikaros CUT&RUN replicates from frozen B cells, showing **A** total mapped reads, **B** total number of called peaks, **C** fraction of reads in peaks (FRiP) scores **D** Genome-wide correlations of reads in 10 kb bins. The overlaps of peak regions for **E** H3K4me3 and **F** Ikaros were compared via binary heatmap, upset, and venn diagrams

**Fig. 3:**
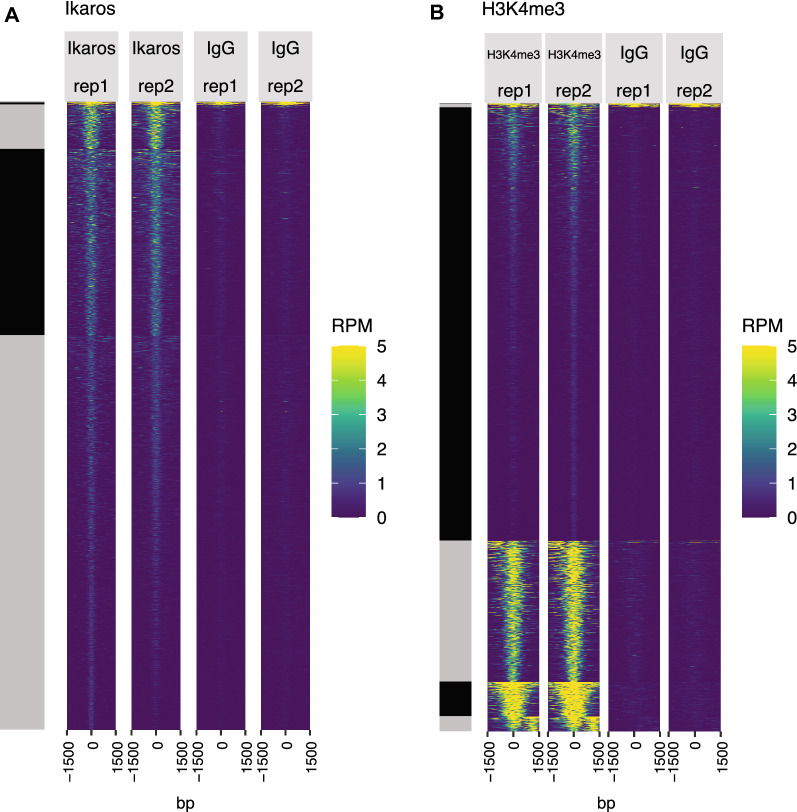
Evaluation of CUT&RUN enrichment profiles with clustered signal heatmap. **A** Heatmap showing the normalized (reads per million (RPM) signal of each CUT&RUN dataset over consensus peak regions for **A** Ikaros replicate datasets (frozen) with IgG controls, or **B** H3K4me3 replicate datasets (frozen) with IgG controls. Clustering was performed with kmeans = 6 for both datasets, and shows an overall similar distribution of signal for called peaks across replicates, exhibiting variable signal across clusters. Also, in either dataset, cluster 1 regions show strong signal in IgG controls and are likely artifacts. These peaks do not map to known blacklisted regions but are associated with heterochromatin. These peaks should be filtered to obtain a final dataset

We used ssvQC to compare the results of Ikaros and H3K4me3 CUT&RUN profiles generated from fresh and frozen B cell samples. The total number of mapped reads ranged from 6—14 million reads per dataset (Additional file 3: Table S1). The number of peaks for H3K4me3 was similar between samples, whereas the number of peaks called for Ikaros differed between datasets (10,809 and 22,022 for fresh and frozen samples, respectively). The FRiP score is defined as the fraction of reads that fall into a peak and is often used as a measure of data quality. Each dataset showed a relatively high FRiP scores, > 0.25 for H3K4me3 and > 0.13 for Ikaros (Additional file 3: Table S1). These scores are in accord with the ENCODE guidelines for ChIP-Seq (FRiP score > 0.1) [4] and are similar to CUT&RUN/CUT&Tag reported scores [11]. Thus, slow freezing of B cells provides equivalent FRiP scores to that of fresh B cells. Next, we performed CUT&RUN for Ikaros and the histone modification H3K4me3 with frozen biological replicates and compared data quality with ssvQC. Overall, the data was consistent between replicates, exhibiting a comparable number of mapped reads, number of peaks, FRiP scores, and Pearson correlation of read counts (Fig. 2A–D). ssvQC was further used to directly compare the peak overlap for each factor, and depicted a moderate degree of overlap across datasets (Fig. 2E, F and Additional file 1: Fig. S1). The data stored by ssvQC is compatible with methods such as ChIPpeakAnno that perform permutation tests to assess statistical significance of peak overlap [12]. Additionally, the strand cross-correlation (SCC) analysis of ssvQC detects the clustering of reads in CUT&RUN dataset independently of peak calling. ssvQC determines the strand-shift profile based on the position of reads on either strand and shifts them towards one another, and then calculates the correlation between the position of reads on the two strands at each progressive shift. Overall, each dataset produced provided a similar SCC score, providing an assessment of data quality independently of peak call (Additional file 2: Fig. S2). Finally, ssvQC can be used to cluster datasets to provide a valuable visual inspection of data quality across genomic regions of interest (Fig. 3).

### Discussion

Despite the increasing application of CUT&RUN for epigenomic profiling, there is not currently any agreement on experimental protocols starting with different types of biological material. Quality control is a critical process when validating these methods. While there are a variety of methods for gathering quality control metrics (library size, alignment rate, read duplication rate, fragment size distribution, *E. coli* aligned read fraction, etc.), including as part of the CUT&RUNTools pipeline, it is still difficult and time consuming to sythesize metrics into an overall view of data quality. Along with numerical metrics, ssvQC provides visualizations of signal enrichment which allow users to make qualitative judgments about their data.

In our experience, fresh biological material from cultured cells or tissues generally provides superior results over frozen material for CUT&RUN. However, when performing lengthy cell isolation procedures such as tissue dissection and cell sorting, and when working with collaborating laboratories at different institutions where shipping of biological material is required, it is not always feasible to proceed directly with sample processing. We therefore wanted to evaluate a slow freezing protocol before performing CUT&RUN. Our analysis indicates that frozen samples serve as a good starting point for performing CUT&RUN for a histone mark and a transcription factor.

To compare the quality of datasets from different sample sources, we developed the ssvQC package to incorporate a set of valuable QC metrics for the evaluation of data quality of CUT&RUN experiments. The ssvQC package reports these metrics in a clear report with understandable graphics. Because ssvQC utilizes the data.table package, it can efficiently process large collections of CUT&RUN, ChIP-seq, ATAC-seq and other sequence enrichment data. Users can reproduce data QC and analysis results in a uniform processing pipeline and integrated software package in R. We performed replicate CUT&RUN from frozen B cells and show features of ssvQC to assess data quality of multiple sequence enrichment datasets.

### Limitations

This study employed an analysis of single fresh and frozen samples, and more replicates and conditions could be incorporated to more rigorously evaluate the optimal experimental conditions.

## Supplementary Information

**Additional file 1: Figure S1**. Analysis of peakset with ssvQC. A) a barplot showing peak counts or each dataset, as well as venn diagrams for H3K4me3 and Ikaros datasets. B) As in A, but peak counts were filtered by qValue scores.

**Additional file 2: Figure S2**. SCC analysis of CUT&RUN data with ssvQC. A) Average strand cross correlation plots for the overlapped peak set in Ikaros datasets (top) or in H3K4me3 datasets (bottom). Blue dashed line indicates correlation maxima and estimated average fragment size. Red dashed line indicates read size. B) The correlation of the libraries estimated fragment size versus at read size for all regions in overlapped peak set. The light red region indicates a correlation at read size >0.9, and peaks that fall within this region are therefore likely artifacts.

**Additional file 3: Table S1**. CUT&RUN quality control on fresh versus frozen samples (XLSX 10 KB)

## Data Availability

The datasets generated and/or analyzed during the current study are available on the NCBI Gene Expression Omnibus database with accession # GSE172130.

## Abbreviations

bp: Base pair
SCC: Strand cross-correlation
H3K4me3: Histone H3 Lysine 4 trimethyl
FRiP: Fraction of reads in peaks
CUT&RUN: Cleavage Under Targets and Release Using Nuclease
ChIP: Chromatin Immunoprecipitation
MNase: Micrococcal nuclease
QC: Quality control
RPM: Reads per million
