# Supplementary material for: ssvQC: an integrated CUT&RUN quality control workflow for histone modifications and transcription factors
Source: BMC Res Notes. 2021 Sep 20;14:366. doi: 10.1186/s13104-021-05781-8 (PMC8454122; doi:10.1186/s13104-021-05781-8)
Supplement: Supplementary file 1 — Additional file 1: Figure S1. Analysis of peakset with ssvQC. A) a barplot showing peak counts or each dataset, as well as venn diagrams for H3K4me3 and Ikaros datasets. B) As in A, but peak counts were filtered by qValue scores. [file 13104_2021_5781_MOESM1_ESM.pdf]

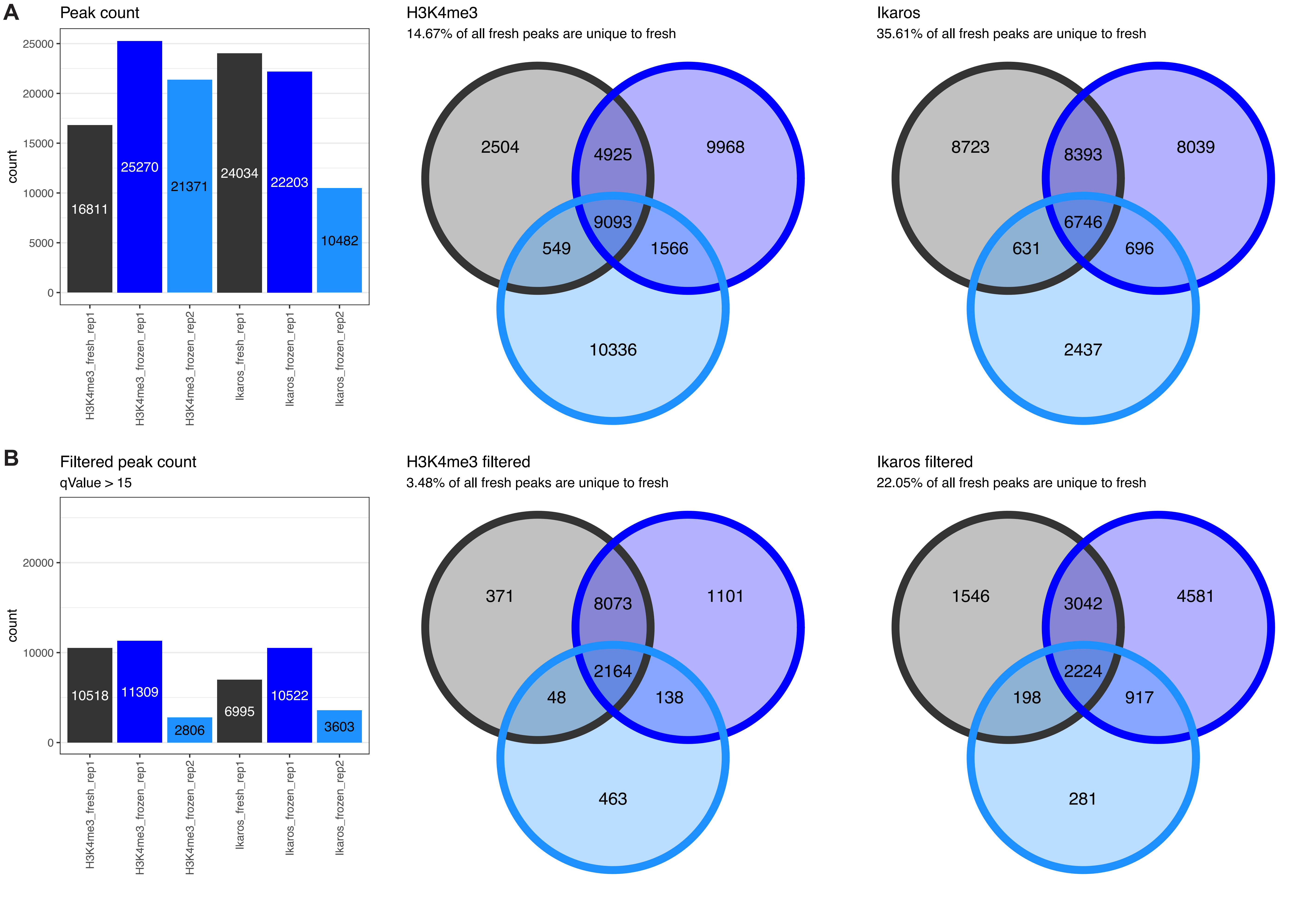
A
Peak count
count
25000
20000
15000
10000
5000
0
16811
25270
21371
24034
22203
10482
H3K4me3_fresh_rep1
H3K4me3_frozen_rep1
H3K4me3_frozen_rep2
Ikaros_fresh_rep1
Ikaros_frozen_rep1
Ikaros_frozen_rep2
H3K4me3
14.67% of all fresh peaks are unique to fresh
2504
4925
9968
9093
549
1566
10336
Ikaros
35.61% of all fresh peaks are unique to fresh
8723
8393
8039
6746
631
696
2437
B
Filtered peak count
qValue > 15
count
20000
10000
0
10518
11309
2806
6995
10522
3603
H3K4me3_fresh_rep1
H3K4me3_frozen_rep1
H3K4me3_frozen_rep2
Ikaros_fresh_rep1
Ikaros_frozen_rep1
Ikaros_frozen_rep2
H3K4me3 filtered
3.48% of all fresh peaks are unique to fresh
371
8073
1101
2164
48
138
463
Ikaros filtered
22.05% of all fresh peaks are unique to fresh
1546
3042
4581
2224
198
917
281
