# Supplementary figures and images for: ssvQC: an integrated CUT&RUN quality control workflow for histone modifications and transcription factors
Source: BMC Res Notes. 2021 Sep 20;14:366. doi: 10.1186/s13104-021-05781-8 (PMC8454122; doi:10.1186/s13104-021-05781-8)

**A**

Ikaros and IgG replicates

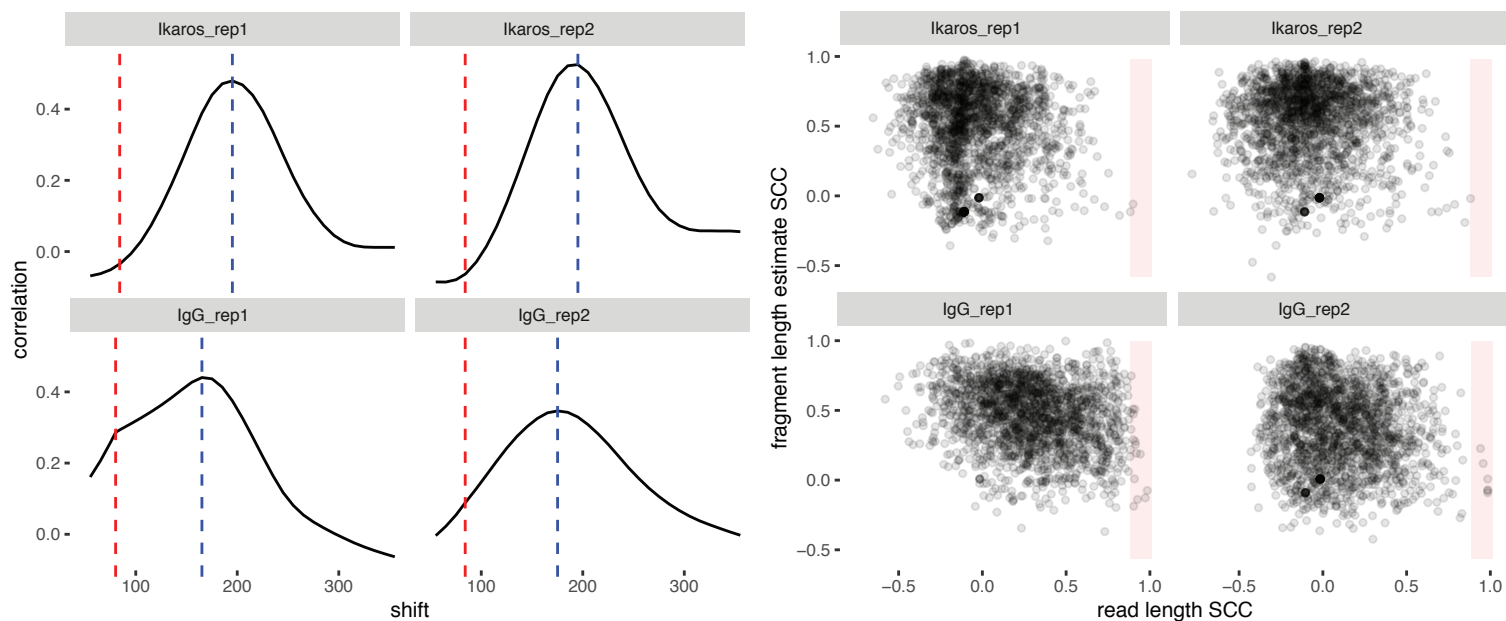**B**

H3K4me3 and IgG replicates

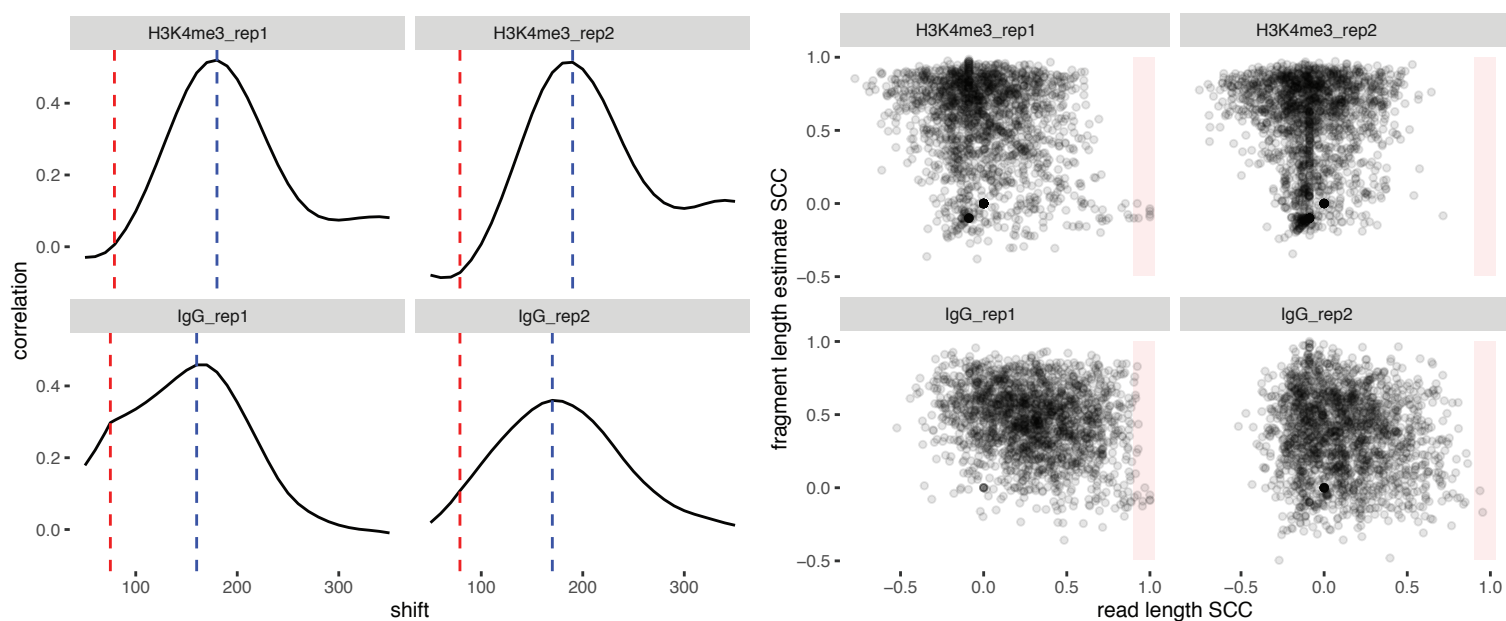

Supplement: Supplementary file 2 — Additional file 2: Figure S2. SCC analysis of CUT&RUN data with ssvQC. A) Average strand cross correlation plots for the overlapped peak set in Ikaros datasets (top) or in H3K4me3 datasets (bottom). Blue dashed line indicates correlation maxima and estimated average fragment size. Red dashed line indicates read size. B) The correlation of the libraries estimated fragment size versus at read size for all regions in overlapped peak set. The light red region indicates a correlation at read size >0.9, and peaks that fall within this region are therefore likely artifacts. [file 13104_2021_5781_MOESM2_ESM.pdf]
